# Pu-erh Tea Inhibits Tumor Cell Growth by Down-Regulating Mutant p53

**DOI:** 10.3390/ijms12117581

**Published:** 2011-11-07

**Authors:** Lanjun Zhao, Shuting Jia, Wenru Tang, Jun Sheng, Ying Luo

**Affiliations:** 1 Lab of Molecular Genetics of Aging & Tumor, Faculty of Life Science and Technology, Kunming University of Science & Technology, Kunming 650224, Yunnan, China; E-Mails: zlj_nwu@163.com (L.Z.); lilith-jia@hotmail.com (S.J.); twr@sina.com (W.T.); 2 Lab of Molecular Genetics of Aging & Tumor, Faculty of Environmental Science and Engineering, Kunming University of Science & Technology, Kunming 650224, Yunnan, China; 3 Molecular Biology Laboratory, Yunnan Agricultural University, Kunming, Yunnan 650201, China; 4 Puer Institute of Pu-erh Tea, Puer 665000, Yunnan, China

**Keywords:** Pu-erh tea, tumor cell growth inhibition, mutant p53

## Abstract

Pu-erh tea is a kind of fermented tea with the incorporation of microorganisms’ metabolites. Unlike green tea, the chemical characteristics and bioactivities of Pu-erh tea are still not well understood. Using water extracts of Pu-erh tea, we analyzed the tumor cell growth inhibition activities on several genetically engineered mouse tumor cell lines. We found that at the concentration that did not affect wild type mouse embryo fibroblasts (MEFs) growth, Pu-erh tea extracts could inhibit tumor cell growth by down-regulated S phase and cause G1 or G2 arrest. Further study showed that Pu-erh tea extracts down-regulated the expression of mutant p53 in tumor cells at the protein level as well as mRNA level. The same concentration of Pu-erh tea solution did not cause p53 stabilization or activation of its downstream pathways in wild type cells. We also found that Pu-erh tea treatment could slightly down-regulate both HSP70 and HSP90 protein levels in tumor cells. These data revealed the action of Pu-erh tea on tumor cells and provided the possible mechanism for Pu-erh tea action, which explained its selectivity in inhibiting tumor cells without affecting wild type cells. Our data sheds light on the application of Pu-erh tea as an anti-tumor agent with low side effects.

## 1. Introduction

Pu-erh tea is produced in the Yunnan province of China. The key process of Pu-erh preparation includes the step of fermentation, in which microorganisms play a very important role in producing the taste, color, fragrances, as well as the functional components. This special preparation process makes Pu-erh tea unique in terms of its shelf life, as well as its bioactivities. As we know, green tea needs to be consumed as fresh as possible, mainly due to the instability of its anti-oxidant components. However, it is believed in Chinese tradition that the taste, as well as the function of Pu-erh tea gets better with increasing shelf time, mainly because the fermentation process is still ongoing during storage. This leads to a very interesting hypothesis, that the bioactivity components of Pu-erh tea are produced during the fermentation process.

Literature researching the chemical components and bioactivities of Pu-erh tea is very limited. It has been shown that the Trolox equivalent antioxidant capacity (TEAC) values of the water extracts of Pu-erh tea (WEPT) is similar to green tea, oolong tea, and black tea, indicating that WEPT had a significant antioxidant activity [1]. However, due to the fermentation and preservation period, the concentration of total catechins in Pu-erh tea is one tenth of that in green tea suggesting the active substances contributing to the antioxidant activity of Pu-erh tea might be formed during fermentation [1]. Another study also found that Pu-erh tea ethyl acetate extract fraction has almost no monomeric polyphenols, theaflavins, and gallic acid, but showed a much stronger antioxidant effect than typical green tea catechin (−)-epigallocatechin-3-gallate [2]. A very recent study isolated a new amide, *N*-(3,4-dihydroxybenzoyl)-3,4-dihydroxybenzamide from the Pu-erh tea and showed it prevented H_2_O_2_-induced cell death of human micro-vascular endothelial cell [3]. However, it has also been shown by HPLC-DAD-MS coupled with 2,2′-azinobis (3-ethylbenzthiazolinesulfonic acid) diammonium salt (ABTS) assay that free radical scavengers in Pu-erh tea still attributed to gallic acid, (−)-gallocatechin, (−)-epigallocatechin, (+)-catechin, caffeine, (−)-epicatechin, (−)-epigallocatechingallate, rutin, (−)-epicatechingallate, quercetin-3-glucoside, and kaempferol-3-glucoside. They proposed that free radical scavenger activity in Pu-erh raw tea was stronger than the ripe one, and the activity decreased with the increase of preservation [4]. A recent study also showed that the raw Pu-erh tea had significantly higher antioxidant activities than the ripened pu-erh teas in the 1,1-diphenyl-2-picrylhydrazyl, Trolox equivalent antioxidant capacity. The epigallocatechin-3-gallate, epigallocatechin, epicatechin-3-gallate, and quinic acid were decreased and gallic acid was increased in a year-dependent manner [5].

In spite of the debates on its anti-oxidant activity, Pu-erh tea has been shown to have anti-obesity and anti-tumor activity. An earlier study showed that high molecular weight fractions of green tea, black tea, oolong tea, and Pu-erh tea induced apoptosis in human monoblastic leukemia U937 cells as well as in stomach cancer MKN-45 cells [6]. It has also been shown that the gains in body weight, levels of triacylglycerol, and total cholesterol were suppressed in Pu-erh tea-treated rats, which correlated with the down-regulation of fatty acid synthase (FAS) expression in the livers of rats fed with tea leaves. *In vitro* study revealed that FAS expression in hepatoma HepG2 cells was suppressed by the extracts of Pu-erh tea at both protein and mRNA levels, which might contribute to the cell growth inhibition [7]. Further study showed the extract of Pu-erh raw tea with ethyl acetate (PR-3) contributed to the most effective hypolipidemic potential and decreased the expression of FAS and inhibited the activity of acetyl-coenzyme A carboxylase by stimulating AMP-activated protein kinase. Moreover, PR-3-5s blocked the progression of the cell cycle at the G1 phase by inducing p53 and p21 expression [8]. Our previous study also showed that Pu-erh tea could cause cell cycle arrest of the human gastric cancer cell line SGC-7901, but not affecting normal CCC-HEL-1 cells [9]. These studies suggested that Pu-erh tea has the potential of anti-tumor activity. However, the mechanism and the molecular targets of its bioactivity are still not clear. Due to the unique procedure during preparation, it is speculated that Pu-erh tea might be different with green tea in terms of its anti-tumor action. The understanding of its mechanism in tumor cell growth inhibition will greatly facilitate its use in cancer prevention.

In this study, we engineered several mouse tumor cell lines by introducing either p53 mutant (p53N236S, p53N239S in human) or/and Ras mutant (H-RasV12) into mouse embryo fibroblasts (MEFs) with *p53^−/−^* genetic background. IARC TP53 database (version R14, November 2009) [10] shows that human p53N239S (or p.N239S) has been reported as a somatic mutation in 32 tumor cases, tumor origin tissues including breast, colon, stomach, hematopoietic and reticuloendothelial systems, liver and intrahepatic bile ducts, bronchus and lung, and brain. The widespread tumor spectrum of p53N239S suggested its importance in tumorigenesis. These engineered cell lines are very clear in terms of their genetic background and the oncogenic proteins they expressed; thus providing us with useful tools in screening and finding the molecular targets of drugs. Because the mutation rates of p53 and Ras are most frequent in human tumors, these tumor cell lines were used to screen anti-tumor activity. Most importantly, we could use the wild type MEFs as a control to test toxicity of drugs.

By using these cell lines with a clear genetic background, we found that water extracts of Pu-erh tea could induce cellular apoptosis in tumor cells but not in control wild type cells. Further study revealed that water extracts of Pu-erh tea could reduce the oncogenic mutant p53 level and thus selectively eliminate the growth advantages of tumor cells.

## 2. Results

### 2.1. The Effect of Pu-erh Tea on Wild Type or Tumor Cells

**Figure 1:**
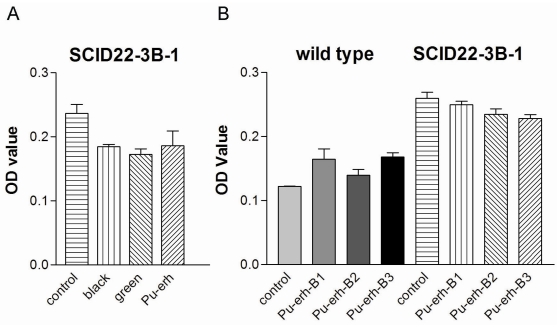
The comparison of tumor cell growth inhibition activity in black tea, green tea, and Pu-erh tea. (**A**) Tumor cell inhibition after 12 h treatment of 0.25 mg/mL water extracts of black tea, green tea, or Pu-erh tea; (**B**) Tumor cell inhibition after 12 h treatment of 0.25 mg/mL water extracts of 3 batches of Pu-erh tea preparation.

First, we tried to compare the action of Pu-erh tea on tumor cells with the action of black tea or green tea, which have been extensively studied. To our surprise, we found that 0.25 mg/mL of water extracts of green tea, black tea or Pu-erh tea have similar activity in inhibiting the growth of tumor cells SCID-3B-1 (Figure 1A). As previously reported, the concentration of total catechins in Pu-erh tea is one tenth that of green tea [1]. The similar activity of green tea and Pu-erh tea in inhibiting tumor cell growth suggested that the tumor cell growth inhibition activity of Pu-erh tea is not due to catechins.

Secondly, we tried to understand whether different batches of Pu-erh tea preparations could affect the tumor cell growth inhibition activity. For this purpose, we used three different batches of Pu-erh tea to treat the tumor cells SCID-3B-1. At the same time, to test whether Pu-erh tea water extracts could act on tumor cells selectively, we used wild type (C57/B6) MEFs as a control for non-specific cellular toxicity of the treatment. The results showed that different batches of Pu-erh tea had very similar activity in tumor cell growth inhibition (Figure 1B, SCID22-3B-1), and the concentration we used (0.25 mg/mL) did not inhibit the growth of wild type cells (Figure 1B, wild type).

**Figure 2:**
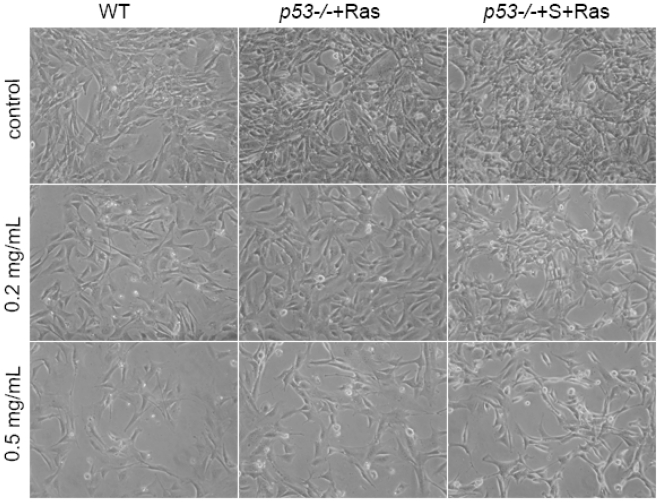
The effect of Pu-erh tea on wild type or tumor cells. The observation of cell morphology change after treating wild type cells or *p53^−/−^*+Ras and *p53^−/−^*+S+Ras tumor cells with serial concentration of Pu-erh tea water extracts for 24 h. Compared with no treatment control, 0.2 mg/mL Pu-erh tea treatment showed some cell deaths in *p53^−/−^*+S+Ras tumor cells, but not in wild type or *p53^−/−^*+Ras tumor cells. The 0.5 mg/mL Pu-erh tea treatment induced obvious cell death in *p53^−/−^*+S+Ras and *p53^−/−^*+Ras tumor cells, also some in wild type cells.

With the control of wild type cells, we treated engineered mouse tumor cell lines *p53^−/−^*+Ras and *p53^−/−^*+S+Ras with Pu-erh tea solution of a serial of concentrations: 0.1 mg/mL, 0.2 mg/mL, 0.3 mg/mL, 0.4 mg/mL and 0.5 mg/mL. After 24 h, we were able to observe cell death in tumor cell lines at the concentration of 0.2 mg/mL (Figure 2, *p53^−/−^*+Ras and *p53^−/−^*+S+Ras column), while wild type cells were not obviously affected (Figure 2, wild type column). Robust cell death was found at the concentration of 0.5 mg/mL (Figure 2, *p53^−/−^*+Ras and *p53^−/−^*+S+Ras column); however, at this concentration cell death was also observed in wild type cells (Figure 2, wild type column). Thus, we used 0.2 mg/mL as the working concentration for selectively killing tumor cells.

### 2.2. Inhibition of Cellular Growth in Several Tumor Cell Lines

To further test the action of Pu-erh tea water extracts on tumor cell growth, three engineered tumor cell lines with different genetic backgrounds were treated with 0.2 mg/mL Pu-erh tea water extracts. Due to the presence of chromatophil components in Pu-erh tea extracts, MTT assay was affected and was not appropriate for testing the action of Pu-erh tea on cell growth. Thus we used crystal violet assay to measure the cell growth curve with or without Pu-erh tea treatment.

**Figure 3:**
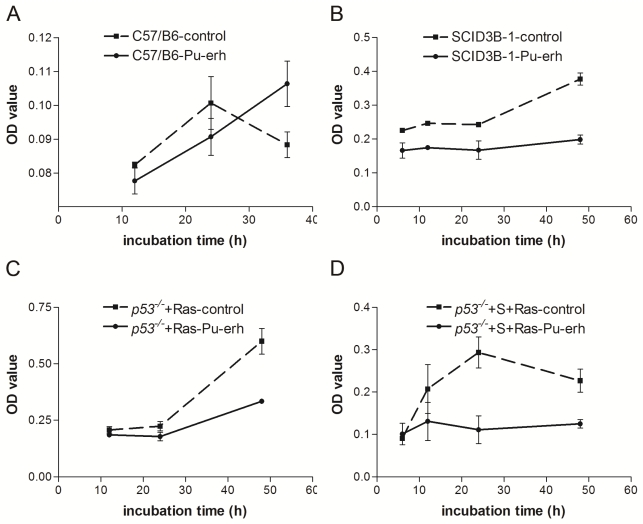
The selective inhibition of cellular growth in several tumor cell lines by 0.2 mg/mL Pu-erh tea treatment. (**A**) Growth curve assay showed that wild type cell growth was not affected; (**B**) The growth of tumor cells SCID22-3B-1were inhibited; (**C**) The growth of tumor cells *p53^−/−^*+Ras were affected; (**D**) The growth of tumor cells *p53^−/−^*+S+Ras were inhibited.

Growth curve assay showed that 0.2 mg/mL Pu-erh tea water extracts could effectively inhibit the growth of the SCID22-3B-1 and *p53^−/−^*+S+Ras tumor cells (Figure 3B,D). 0.2 mg/mL Pu-erh tea water extracts could also inhibit cell growth of the *p53^−/−^*+Ras tumor cells, (Figure 3C), but not as dramatically as the *p53^−/−^*+S+Ras tumor cells. The same concentration of Pu-erh tea water extracts did not affect the growth of the wild type cells (Figure 3A). These results confirmed that 0.2 mg/mL Pu-erh tea water extracts could selectively inhibit the growth of tumor cells, but not affect the wild type cells. The preference of inhibition on SCID22-3B-1 and *p53^−/−^*+S+Ras tumor cells with mutant p53-p53S implied that mutant p53 could be a molecular target of Pu-erh tea action.

### 2.3. Cellular Response of Tumor Cell Lines to Pu-erh Tea Treatment

**Figure 4:**
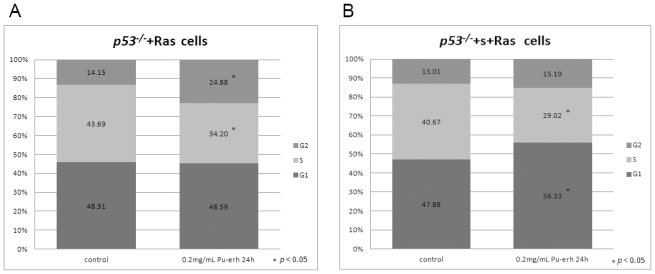
Cell cycle analysis of tumor cell lines at 24 h after 0.2 mg/mL Pu-erh tea treatment. (**A**) Flow cytometry assay of *p53^−/−^*+Ras tumor cells after Pu-erh tea treatment showed that the *p53^−/−^*+Ras tumors cells in S phase were reduced, and those in G2 phase were increased (*p* < 0.05); (**B**) The *p53^−/−^*+S+Ras tumors cells in S phase were reduced, and those in G1 phase were increased (*p* < 0.05).

To further understand the inhibition of tumor cell growth by Pu-erh tea, we performed flow cytometry to analyze the cell cycle regulation in tumor cells after Pu-erh tea treatment. After treating with 0.2 mg/mL Pu-erh tea solution for 24 h, the *p53^−/−^*+Ras and *p53^−/−^*+S+Ras tumors cells were harvested for flow cytometry analysis. The results showed that in Pu-erh tea treated *p53^−/−^*+Ras cells, the proportion of cells in S phase was significantly decreased and that in G2 phase was significantly increased, suggesting a G2 arrest induced by Pu-erh tea treatment in *p53^−/−^*+Ras cells (Figure 4A). Interestingly, in *p53^−/−^*+S+Ras cells, Pu-erh tea caused a significant decrease of cells in S phase and an increase of cells in G1 phase, suggesting a G1 arrest (Figure 4B). No obvious apoptosis cell populations were observed in both cell lines at this concentration. These results showed the cell cycle arrest effect caused by 0.2 mg/mL Pu-erh tea and further confirmed the observation of growth curve assay.

### 2.4. Molecular Response to Pu-erh Tea Treatment in Tumor Cell Lines

**Figure 5:**
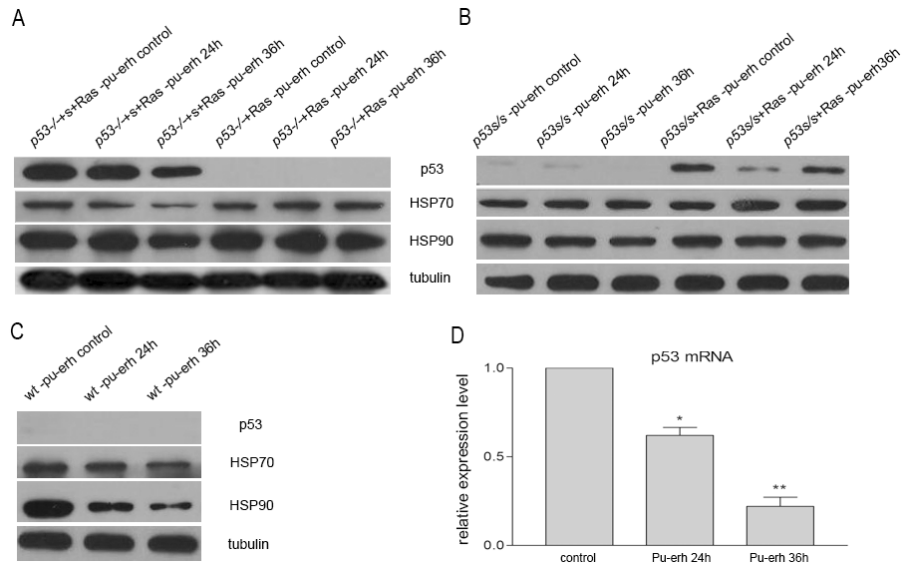
The regulation of p53, HSP70 and HSP90 proteins in tumor cells or wild type cells after 0.2 mg/mL Pu-erh tea water extract treatment. (**A**) The mutant p53 proteins in the *p53^−/−^*+S+Ras tumor cells were down-regulated after treatment for 24 h or 36 h, while the HSP70 and HSP90 protein levels slightly decreased. In *p53^−/−^*+Ras tumor cells with no p53 mutant, HSP70 and HSP90 protein levels were not obviously changed; (**B**) In *p53^S/S^* and *p53^S/S^*+Ras cells, p53S proteins were also down-regulated but not HSP70 and HSP90 protein levels; (**C**) Wild type p53 protein level did not elevate after Pu-erh tea treatment while HSP70 and HSP90 protein levels were decreased; (**D**) Real-time PCR showed that p53 mRNA level in *p53^−/−^*+S+Ras tumor cells was down-regulated by Pu-erh tea treatment.

To understand the molecular basis of Pu-erh tea action on tumor cell cycle arrest and to test whether mutant p53 is the molecular target for Pu-erh tea action, the *p53^−/−^*+S+Ras and *p53^−/−^*+Ras tumor cells were treated with 0.2 mg/mL Pu-erh tea water extracts and harvested at different time points to check the molecular response to Pu-erh tea. Our data showed that in *p53^−/−^*+S+Ras tumor cells, after 0.2 mg/mL Pu-erh tea water extracts treatment, mutant p53 protein level was down-regulated (Figure 5A, p53). We also observed a slight decrease of HSP70 and HSP90 protein level in response to Pu-erh tea treatment (Figure 5A, HSP70, HSP90). These protein regulations, especially the down-regulation of mutant p53 might contribute to the inhibition of cell growth. In *p53^−/−^*+Ras tumor cells, which is null background for p53 and have no mutant p53 expression, we did not observe obvious change of HSP70 or HSP90 after Pu-erh tea treatment (Figure 5A).

To further confirm the Pu-erh tea action on p53S, we used the *p53^S/S^* and *p53^S/S^*+Ras cells which express mutant p53S at a level lower than the *p53^−/−^*+S+Ras tumor cells. After treated cells with 0.2 mg/mL Pu-erh tea water extracts, the p53S protein also decreased in both *p53^S/S^* and *p53^S/S^*+Ras cells (Figure 5B, p53). HSP70 and HSP90 protein level did not show obvious change in both cells (Figure 5B, HSP70, HSP90).

On the other hand, our data also showed that 0.2 mg/mL Pu-erh tea treatment did not up-regulate the wild type p53 level, suggesting the treatment did not initiate stress response in the wild type cells (Figure 5C, p53). Interestingly, the HSP70 and HSP90 protein levels decreased in the wild type cells (Figure 5C, HSP70, HSP90). These data further confirmed the selective inhibition effect of Pu-erh tea on tumor cells.

We did not observe obvious changes in Bax and Bcl-2 protein levels, suggesting that the apoptosis process is not dramatically activated (data not shown). We also did not detect the expression of cell cycle regulator p21 protein in these tumor cells even after Pu-erh tea treatment, suggesting that the downstream of p53 pathway was not activated in these p53 function deficient cells.

To understand the regulation of mutant p53 by Pu-erh tea treatment, we performed real-time PCR to detect p53 mRNA in *p53^−/−^*+S+Ras cells after Pu-erh tea treatment. We found the mutant p53 mRNA level decreased gradually after Pu-erh tea treatment (Figure 5D), which explained the down-regulation of mutant p53 revealed by Western blot 5A). Together these data suggest that Pu-erh tea targets the p53 mutant and inhibits cell growth. Our data also explained the mechanism for the selective effect of Pu-erh tea on tumor cells bearing p53 mutant.

## 3. Discussion

The toxicity of chemotherapeutic drugs has become one of the major issues in chemotherapy. The new developments in cancer fields have focused more and more on cancer prevention, early diagnosis and personalized medicine. Thus, chemicals which could target tumor specific molecules and selectively kill tumor cells become one of the most popular topics in drug screening.

In this study, using the wild type control and genetically engineered tumor cell lines as our screening system, we could easily differentiate the toxicity of drug treatment to normal cells from the tumor cell growth inhibition activity. Furthermore, the specific genetic background provided advantages in identifying the molecular target of the treatment.

Green tea has been reported extensively as anti-tumor reagents, which mainly attributes to its anti-oxidant components, such as EGCG, *etc*., and multiple tumorigenic proteins were found to be regulated by EGCG at different concentrations. Among those, tumor promoting factors like DNA methyltransferase (DNMT), epidermal growth factor receptor (EGFR), hepatocyte growth factor receptor (HGFR), insulin-like growth factor 1 receptor (IGF1R), matrix metalloproteinase (MMP), vascular endothelial growth factor A (VEGFA) and BCL-2 are down-regulated by EGCG while tumor suppressor p53 is up-regulated [11]. These mechanisms provided the basis for the selective inhibition of tumor growth by green tea while not affecting wild type cells.

Due to its special fermented processing and the incorporation of microbes, Pu-erh tea might be different from green tea in terms of its tumor cell growth inhibition activity, as well as its molecular targets. Also, the fermentation by microbes might increase the bioavailability of Pu-erh tea components. Nevertheless, as a regular drink in human life, we speculated that the toxicity of Pu-erh tea to normal cells should be very limited. It has been also reported that the safe dosage for Sprague-Dawley and Wistar rats could be 5000 mg/kg/day [12,13].

In this study, we showed that Pu-erh tea has comparable tumor cell growth inhibition activity with black tea and green tea. However, several independent groups have shown that the fermentation process of Pu-erh tea could reduce its total catechins and related anti-oxidant activity [1,4,5]. It is reasonable to speculate that the tumor cell growth inhibition activity of Pu-erh tea is due to other natural compounds. Further studies are ongoing in finding the natural compound(s) responsible for its bioactivity.

We also found that Pu-erh tea water extracts selectively inhibit tumor cell growth at a concentration that does not affect wild type cells. At this concentration, Pu-erh tea water extracts inhibited tumor cell growth by down-regulated S phase and caused G1 or G2 arrest. This selective inhibition of tumor cells would greatly reduce the non-specific cyto-toxicity and provide a way of cancer prevention or treatment that potentially helps to solve the current problem of cyto-toxicity of most conventional chemotherapy drugs.

Furthermore, our studies revealed that Pu-erh tea water extracts down-regulated the expression of p53 mutant at both mRNA and protein levels. The down-regulation of mutant p53 by Pu-erh tea might eliminate the growth advantage of tumor cells with mutant p53. As we know, more than 50% of human tumors have p53 mutation, and mutant p53 proteins could gain oncogenic function and impact tumor progression and metastasis [14]. Thus, targeting mutant p53 has become one important strategy in drug screening and personalized cancer treatment [15]. Because of its regulation on mutant p53, Pu-erh tea has a potential in cancer treatment with low side effects.

HSP70 and HSP90 proteins have been targeted for cancer therapy [16,17] due to their high expression level in tumor cells and their chaperon characteristics for other oncogenic proteins, e.g., mutant p53 [18]. Here we also found that Pu-erh tea treatment could slightly down-regulate both HSP70 and HSP90 protein levels in tumor cells. However, the down-regulation of HSP70 and HSP90 by Pu-erh tea seemed to depend on genetic background since we did not observe obvious changes of HSP70 and HSP90 in cells with lower p53S or null p53 expression, *i.e*., *p53^S/S^* cells, *p53^S/S^* +Ras cells and *p53^−/−^*+Ras cells.

On the other hand, our data showed that the same concentration of Pu-erh tea solution did not cause p53 stabilization or activation of its downstream pathways in the wild type cells. Growth curve analysis also did not show any growth inhibition of wild type cells by Pu-erh tea treatment. Altogether our data revealed the action of Pu-erh tea on tumor cells and provided a possible mechanism for Pu-erh tea’s selectivity in inhibiting tumor cells without affecting wild type cells.

## 4. Experimental Section

### 4.1. Preparation of Pu-erh Tea Water Extracts

The dry powder form of water extracts of ripe Pu-erh tea was prepared by a standardized protocol. Briefly, 10 g ripe Pu-erh tea leaves were boiled in water for 30 min for three times. The supernatant was collected, concentrated and spray dried to powder form and stored at 4 °C. The powder was resolved in 1 × PBS at 55 °C to make 20 mg/mL stocking solution of Pu-erh tea water extracts. The solution was filtered once with filter paper and once with a syringe 0.2 μm filter (Millipore, MA, USA). The sterilized Pu-erh tea solution was aliquoted and stored at −20 °C. The black tea and green tea water extracts have been made with the same procedure.

### 4.2. Cell Lines, Constructs and Antibodies

Wild type MEFs were harvested and prepared from individual day 13.5 wild type C57/B6 mouse embryos. Wild type MEFs were used for experiments before passage 10 and the cells were passed at the ratio of 1:2 or 1:3 so that the cells would not be diluted and senesced quickly.

Immortalized cell lines 395-3B-1 harboring a p53 point mutation (p53S) were established as described [19]. 395-3B-1 cells immortalized from MEFs double knocked out telomerase and Werner (*mTR^−/−^Wrn^−/−^*) were injected subcutaneously into SCID mouse. The tumors growing from the injected cells were harvested and the tumor cells were cultured to establish SCID22-3B-1 cell line. Sequencing of p53 cDNA from these tumor cells revealed a p53S mutation. Thus the genetic background of SCID22-3B-1 tumor cell line is *mTR^−/−^Wrn^−/−^* *p53^S/S^*.

pBabe-H-RasV12 construct and either empty vector PQCXIH or PQCXIH-p53S construct (as described previously [20]) were introduced into *p53^−/−^* MEF. After 1–2 months antibiotics selection, cell colonies were picked up, expression of p53S or H-RasV12 was analyzed by both Western blot and immunostaining. By this way, *p53^−/−^* MEFs stably introduced both p53S and H-RasV12 (*p53^−/−^*+S+Ras MEFs), or H-RasV12 and empty PQCXIH vector (*p53^−/−^*+Ras MEFs) were established. MEFs were cultured and injected into SCID mice to get *p53^−/−^*+S+Ras and *p53^−/−^*+Ras tumor cells.

*p53^S/S^* and *p53^S/+^* MEFs were harvested and prepared from individual day 13.5 embryos from *p53^S/+^* mice. pBabe-H-RasV12 construct were introduced into these cells to establish *p53^S/S^*+Ras or *p53^S/+^*+Ras MEFs.

All cell lines were cultured in DMEM media supplemented with 10% fetal bovine serum (Hyclone, CA, USA) in a 3% oxygen and 5% CO2 incubator at 37 °C.

Antibodies used for Western blot were anti-p16Ink4a (M-156) (1:500, Santa Cruz, CA, USA), anti-p21 (F-5) (1:500, Santa Cruz, CA, USA), anti-p53 Ab1(clone PAb240) (1:250, Neomarker, CA, USA), anti-phospho-p53 (Ser15) (1:500, Cell Signaling, MA, USA), anti-Hsp70 (1:1000, Stressgen, BC, Canada), anti-Hsp90 (1:1000, Stressgen, BC, Canada), anti-γ-tubulin (1:5000, Upstate, NY, USA).

### 4.3. Growth Curve Assay

2.5 × 104 cells were seeded per well in 12 well plates, triplicates were prepared for each sample. At each time point, cells were fixed for 20 min at room temperature in 10% buffered formalin, and stained with 1 mL 0.1% crystal violet for 20 min at room temperature. After washing and drying the plates, cells were treated with 2 mL 10% acetic acid and OD of the extracted dye was measured at 595 nm.

### 4.4. Real-Time RT-PCR

RNA was extracted from cells and cDNA was synthesized by reverse transcription. Real-time PCR was performed using SYBR-Green PCR master mix following the manufacturer’s instruction (Applied Biosystems, CA). The primers used are as following: p53 (Forward primer: 5′-TGATGGAGAGTATTTCACCC-3′, Reverse primer: 5′-GGGCATCCTTTAACTCTAAG-3′). β-actin (Forward primer: 5′-AGAGGGAAATCGTGCGTGAC-3′, Reverse primer: 5′-CAATAGTGATGACCTGGCCGT-3′).

### 4.5. Cell Cycle Analysis

Cell cycle analysis was done by flow cytometry (BD, FACS Vantage SE). Data were analyzed by Flowjo. Percentages of cells in G1, S, and G2 were determined using the Dean-Jett-Fox algorithm.

## 5. Conclusions

Our study found that Pu-erh tea extracts could inhibit tumor cell growth by down-regulating S phase and G1 or G2 arrest at a concentration that does not affect wild type cell growth. We showed that Pu-erh tea extracts down-regulated the mutant p53. HSP70 and HSP90 were regulated depending on the cellular genetic background. These data revealed the action of Pu-erh tea on tumor cells, providing the possible mechanism for Pu-erh tea action, and suggested the possible application of Pu-erh tea in cancer treatment.
